# Endogenous Antibody Responses to SARS-CoV-2 in Patients With Mild or Moderate COVID-19 Who Received Bamlanivimab Alone or Bamlanivimab and Etesevimab Together

**DOI:** 10.3389/fimmu.2021.790469

**Published:** 2021-12-09

**Authors:** Lin Zhang, Josh Poorbaugh, Michael Dougan, Peter Chen, Robert L. Gottlieb, Gregory Huhn, Stephanie Beasley, Montanea Daniels, Thi Ngoc Vy Trinh, Melissa Crisp, Joshua Joaquin Freitas, Peter Vaillancourt, Dipak R. Patel, Ajay Nirula, Nicole L. Kallewaard, Richard E. Higgs, Robert J. Benschop

**Affiliations:** ^1^ Lilly Research Laboratories, Eli Lilly and Company, Indianapolis, IN, United States; ^2^ Massachusetts General Hospital and Harvard Medical School, Boston, MA, United States; ^3^ Department of Medicine, Women’s Guild Lung Institute, Cedars-Sinai Medical Center, Los Angeles, CA, United States; ^4^ Department of Internal Medicine, Center for Advanced Heart and Lung Disease, Baylor University Medical Center, Dallas, TX, United States; ^5^ Baylor Scott & White Research Institute, Dallas, TX, United States; ^6^ The Ruth M. Rothstein CORE Center, Cook County Health, Chicago, IL, United States

**Keywords:** serology, antibodies, immune response, COVID-19, bamlanivimab, etesevimab

## Abstract

**Background:**

Neutralizing monoclonal antibodies (mAbs) to SARS-CoV-2 are clinically efficacious when administered early, decreasing hospitalization and mortality in patients with mild or moderate COVID-19. We investigated the effects of receiving mAbs (bamlanivimab alone and bamlanivimab and etesevimab together) after SARS-CoV-2 infection on the endogenous immune response.

**Methods:**

Longitudinal serum samples were collected from patients with mild or moderate COVID-19 in the BLAZE-1 trial who received placebo (n=153), bamlanivimab alone [700 mg (n=100), 2800 mg (n=106), or 7000 mg (n=98)], or bamlanivimab (2800 mg) and etesevimab (2800 mg) together (n=111). A multiplex Luminex serology assay measured antibody titers against SARS-CoV-2 antigens, including SARS-CoV-2 protein variants that evade bamlanivimab or etesevimab binding, and SARS-CoV-2 pseudovirus neutralization assays were performed.

**Results:**

The antibody response in patients who received placebo or mAbs had a broad specificity. Titer change from baseline against a receptor-binding domain mutant (Spike-RBD E484Q), as well as N-terminal domain (Spike-NTD) and nucleocapsid protein (NCP) epitopes were 1.4 to 4.1 fold lower at day 15-85 in mAb recipients compared with placebo. Neutralizing activity of day 29 sera from bamlanivimab monotherapy cohorts against both spike E484Q and beta variant (B.1.351) were slightly reduced compared with placebo (by a factor of 3.1, p=0.001, and 2.9, p=0.002, respectively). Early viral load correlated with the subsequent antibody titers of the native, unmodified humoral response (p<0.0001 at Day 15, 29, 60 and 85 for full-length spike).

**Conclusions:**

Patients with mild or moderate COVID-19 treated with mAbs develop a wide breadth of antigenic responses to SARS-CoV-2. Small reductions in titers and neutralizing activity, potentially due to a decrease in viral load following mAb treatment, suggest minimal impact of mAb treatment on the endogenous immune response.

## Introduction

Coronavirus disease 2019 (COVID-19) is caused by the novel human pathogen severe acute respiratory syndrome coronavirus 2 (SARS-CoV-2) and has resulted in widespread global morbidity and mortality (1).

The host immune response continues to be the best defense against SARS-CoV-2 (2). While both innate and adaptive immune processes are important, the humoral response against the virus remains critical (3, 4). Healthy individuals exposed to SARS-CoV-2 mount a robust immune response involving the production of anti-SARS-CoV-2 antibodies against a wide variety of SARS-CoV-2 epitopes across the nucleocapsid protein (NCP) and the spike protein (5). Virus-neutralizing antibodies are primarily directed to the receptor-binding domain (RBD) of the spike protein, however some neutralizing epitopes reside within the N-terminal domain (NTD) (6, 7). While individuals who recover from COVID-19 develop robust immunoglobulin G antibody responses against SARS-CoV-2 that can persist for at least 3-5 months after infection (8, 9), waning titers and plasma neutralizing abilities over time have been reported (10–12).

Several neutralizing monoclonal antibodies (mAbs) have been developed to treat COVID-19. Two such antibodies, bamlanivimab and etesevimab, bind to the RBD region of the spike protein and have been shown to reduce nasopharyngeal viral load in patients with mild or moderate COVID-19 and prevent progression of COVID-19 leading to hospitalization or death (13, 14). The efficacy of these mAbs can be reduced by mutations within the RBD spike protein, such as at residue E484, which negatively impacts bamlanivimab binding (13, 15).

Studies are necessary to assess the potential effect treatment with neutralizing mAbs has on the specificity, magnitude, and duration of the endogenous antibody response to SARS-CoV-2 infection. Using serum samples collected from patients with mild or moderate COVID-19 enrolled in the BLAZE–1 trial who received placebo, bamlanivimab alone, or bamlanivimab and etesevimab together, we performed longitudinal analyses of antibody responses to SARS-CoV-2 infection. We examined the binding and neutralization activity of sera to SARS-CoV-2 viral proteins and assessed the relationship between early viral load and antibody titers.

## Materials and Methods

### Convalescent Serum Samples

Samples were obtained from individuals infected with SARS-CoV-2 who received placebo, bamlanivimab (700, 2800, or 7000 mg), or bamlanivimab (2800 mg) and etesevimab (2800 mg) together in the phase 2 portion of the BLAZE-1 trial (NCT04427501) as described previously (13). All donors provided written informed consent. Treatment was administered within three days of the first positive SARS-CoV-2 test sample collection. Serum samples were collected longitudinally at time of enrollment [baseline (prior to infusion)] and on day 3, day 15, day 29, day 60, and day 85 after infusion. Prior to use in each assay, serum samples were centrifuged for 5 minutes at 10000 x *g* to pellet any debris.

### Luminex Multiplexing

Luminex xMAP technology is an established, multiplex, flow cytometry-based platform that allows the simultaneous quantitation of many protein analytes in a single reaction (16). Antigen-coated microspheres were used to detect and quantitate endogenous antibodies against multiple viral proteins simultaneously (**Table 2**). The method was performed essentially as previously described (17). Briefly, patient serum samples were titrated (1:800 – 1:8E9) in phosphate buffered saline-high salt solution (PBS-HS; 0.01 M PBS, 1% [bovine serum albumin] BSA, 0.02% Tween, 300 mM NaCl) and combined with Luminex MAGPlex microspheres coupled with either SARS-CoV-2 or RBD mutant proteins. Diluted serum samples and microsphere solution were incubated for 90 minutes at room temperature, followed by a 60-minute incubation with the detector phycoerythrin-conjugated anti-IgG Fc-specific antibody (#109-115-098, Jackson Labs). Washed beads were then resuspended in a PBS-1% BSA solution and read using a Luminex FlexMAP 3D System with xPONENT Software.

### Pseudovirus Production and Characterization

E484K and E484Q mutagenesis reactions were performed using the QuickChange Lightning Site-Directed Mutagenesis Kit (Agilent #210519) using a template of a spike mammalian expression vector based on the Wuhan sequence (Genbank MN908947.3) with a deletion of the C-terminal 19 amino acids. For the beta variant (B.1.351) pseudovirus a consensus sequence representative of lineage was synthesized and incorporated by Gibson cloning. Pseudoviruses bearing mutant spike proteins were produced using the delta-G-luciferase recombinant Vesicular Stomatitis Virus (rVSV) system (KeraFast EH1025-PM, Whitt 2010). Briefly, 293T cells were transfected with individual mutant spike expression plasmids, and 16-20 hours later, transfected cells were infected with VSV-G-pseudotyped delta-G luciferase rVSV, and 16-20 hours thereafter conditioned culture medium was harvested, clarified by centrifugation at 1320 g for 10 minutes at 4°C, aliquoted and stored frozen at -80°C. Relative luciferase reporter signal read-out was determined by luciferase assay (Promega E2650) of extracts from VeroE6 cells infected with serially-diluted virus. Luciferase activity was measured on a PerkinElmer EnVision 2104 Multilabel Reader.

### Pseudovirus Neutralization Assays

Neutralization assays were carried out essentially as described previously (18). Serum antibodies were diluted 4-fold in negative serum and 10-point 3-fold titrations in 25% negative serum were performed in 384 well polystyrene plates in duplicate using a Beckman (Biomek i5) liquid handler. Positive and negative control antibodies and an unrelated control (hIgG1 isotype) were tested in a 10-point, 3-fold serial dilution starting at 8 µg/mL, 2 µg/mL and 8 µg/mL, respectively, in 25% negative serum. An empirically pre-determined fixed amount of pseudovirus (Wuhan, E484Q, E484K, or the B.1.351 spike) was dispensed by WDII liquid dispenser on titrated serum antibodies and controls and pre-incubated for 20 minutes at 37°C. Following pre-incubation, the virus-antibody complexes were transferred by Biomek i5 to 8,000/well VeroE6 cells in white, opaque, tissue culture treated 384W plates, and incubated for 16-20 hours at 37°C. Control wells included virus only (no antibody; 14 replicates) and cells only (14 replicates). Following infection, cells were lysed with Promega BrightGlo and luciferase activity was measured on the Biotek Synergy Neo2 Multimode Reader.

### Viral Load Determination

Viral load was measured by nasopharyngeal swab followed by quantitative RT-PCR reaction (13, 19). Viral load data is based on the cycle threshold and calculated as an arbitrary unit. The primer sequences for the RT-PCR assay have been reported previously (20).

### Statistical Analysis

Titer is commonly defined as the smallest dilution above the cut point or the dilution factor at the cut point based on an interpolation of assay values that straddle the cut point (21). In the serology assay, we used the latter method to calculate the titers (cut point for full-length spike, spike-RBD, spike-NTD, and NCP was set as 3, and cut point for spike-RBD E484Q was set as 1000). If the maximum signal of a titration curve is less than the cut point, then the titer is imputed as 800 (smallest dilution). The samples were run in three batches in the Luminex serology assay, and batch effect was included as a fixed effect in the statistical model for downstream analysis.

The treatment effects on titers were compared based on change from baseline in log_10_ titer at different time points. Mixed-model repeated-measure analysis with unstructured covariance matrix (2-sided test with α level of 0.05) was used to conduct the significance testing. Treatment group, visit day, treatment × visit interaction, and batch effect were included as fixed effects in the model. Adjustments for multiple testing were not conducted; therefore, the findings should be interpreted as exploratory. The statistical analyses were performed with R software (version 4.0.3) (22). Spearman correlations between viral load (at baseline and visit day 3) and log_10_ titer fold change from baseline (at visit day 3, 15, 29, 60, and 85) were computed. Since most samples from the same patient were processed in the same batch, batch has minimum impact on this correlation.

To calculate IC50 titer of data from the pseudovirus neutralization assay, a 4-parameter logistic function was used to estimate the absolute IC50 based on 1/dilution factor (bottom is fixed at 0). If a sample has less than 50% neutralization over observed concentration range or a poor fit (the standard error of the IC50 is not estimable, majority of which has less than 50% neutralization over observed concentration range, or the estimated IC50 is larger than the maximum 1/dilution factor), its IC50 titer was imputed to 0.125 (twice the maximum 1/dilution factor). For the pseudovirus neutralization assay analysis, treatment effects (compared to placebo) were compared based on log_10_ 1/IC50 titer using a non-parametric Steel’s Test using JMP^®^ (v14.1).

## Results

### Patient Characteristics

Serum samples were obtained from patients with mild or moderate COVID-19 enrolled in the BLAZE–1 trial who received placebo, bamlanivimab (700 mg, 2800 mg, or 7000 mg), or bamlanivimab (2800 mg) and etesevimab (2800 mg) together. Baseline demographics and clinical characteristics of patients enrolled in the BLAZE-1 study have previously been reported (13). Among the placebo cohort, no patient reported an immunocompromised condition and 3 patients (1.96%) reported receiving immunosuppressive treatment at baseline, while among recipients of bamlanivimab alone or bamlanivimab and etesevimab together 6 patients (1.45%) reported immunocompromised condition and 10 patients (2.41%) reported receiving immunosuppressive treatment at baseline. A total of 568 patients provided serum samples, 560 samples were collected at baseline, and postbaseline samples were collected at days 3, 15, 29, 60, and 85 (**Table 1**). Patients had mild to moderate COVID-19, defined per US Food and Drug Administration guidance (23), with symptoms including but not limited to fever, cough, sore throat, malaise, headache, muscle pain, gastrointestinal symptoms, or shortness of breath with exertion. A total of 440 patients (77.5%) had mild COVID-19 at baseline, while 128 (22.5%) had moderate COVID-19 at baseline. Means of viral load at baseline were 6.3 (standard deviation [SD] 2.2) and 6.9 (SD 2.0) for patients with mild and moderate COVID-19, respectively. Participants were recruited into the study during the summer of 2020, prior to the widespread emergence of many of the SARS-CoV-2 variants of interest/concern such as the Alpha, Beta, Gamma, and Delta variants. Genotypic analysis of the SARS-CoV-2 virus present in baseline samples confirm absence of these SARS-CoV-2 variants in this cohort, with the majority of infecting viruses containing the D614G substitution in spike found in the B.1 pangolin lineages.

**Table 1:** Number of patients in the BLAZE-1 study that provided serum samples at each timepoint.

Treatment	Baseline	Day 3	Day 15	Day 29	Day 60	Day 85
Placebo	152	140	124	126	125	120
Bamlanivimab 700 mg	99	93	90	86	82	84
Bamlanivimab 2800 mg	104	100	90	91	91	92
Bamlanivimab 7000 mg	97	91	89	86	77	80
Bamlanivimab (2800 mg) and etesevimab (2800 mg) together	108	98	95	96	91	80

Patients received placebo, bamlanivimab alone (700, 2800, or 7000 mg), or bamlanivimab (2800 mg) and etesevimab (2800 mg) together.

### Antibody Responses to SARS-CoV-2

**Figure 1:**
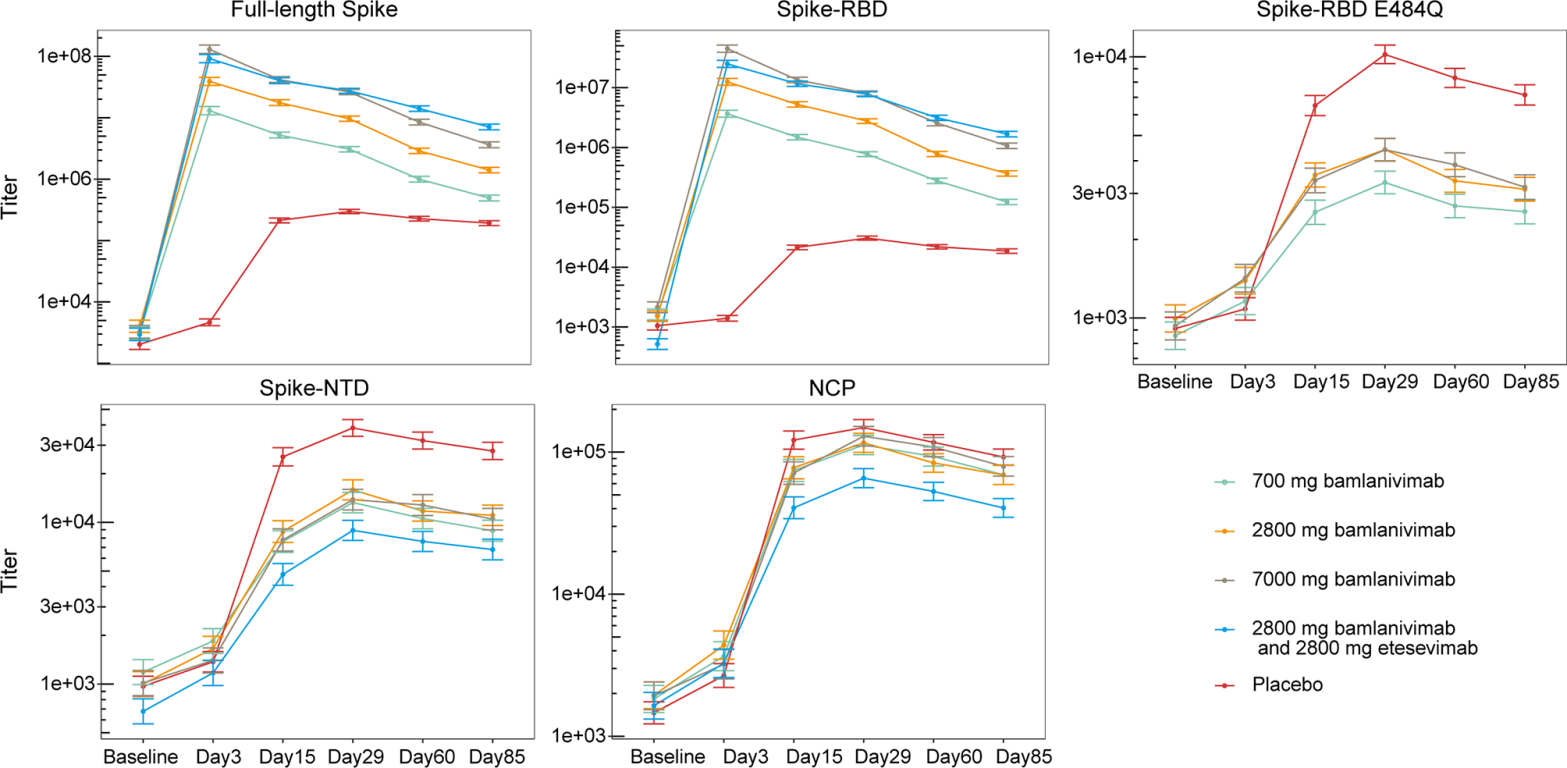
Antibody responses to SARS-CoV-2 viral proteins among patients treated with bamlanivimab monotherapy, bamlanivimab and etesevimab together, and placebo. Least squares means ( ± SE) were plotted across visit days for different treatment groups. The full-length spike protein carries the D614G substitution. Titers against Spike-RBD E484Q not shown for cohort receiving bamlanivimab and etesevimab together as etesevimab binds to this mutant protein. The number of samples at each timepoint are outlined in **Table 1**. RBD, Receptor binding domain; NCP, Nucleocapsid protein; NTD, N-terminal domain; SE, Standard error.

A multiplex assay using the Luminex platform was performed to determine the magnitude and specificity of antibody responses to SARS-CoV-2. Antibody titers against four different SARS-CoV-2 spike versions (the full-length spike protein bearing the D614G substitution, the Spike-RBD, the RBD carrying the E484Q alteration, or the NTD) and the nucleocapsid protein (NCP) (**Table 2**) were calculated using serum samples obtained from each cohort. Since bamlanivimab does not bind significantly to Spike RBD with alterations at residue E484 (15, 24), and as the epitopes for bamlanivimab and etesevimab lie within the spike RBD (25, 26), titers against Spike RBD E484Q, NTD, or NCP proteins solely reflect the endogenous antibody response. Titers against the full-length spike protein and the Spike-RBD, were greater among cohorts that received bamlanivimab monotherapy (all doses), as anticipated (27), and bamlanivimab and etesevimab together compared with the placebo cohort, reflecting detection of bamlanivimab and/or etesevimab (**Figure 1**). Tracking the endogenous antibody responses against SARS-CoV-2 proteins, titers were generally lowest at baseline, with levels increasing over time and peaking around day 29 followed by slight declines in titers through day 85. The same patterns for Spike RBD E484Q, the NTD, and NCP titers were observed in the mAb-receiving cohorts. In the placebo cohort, least square means of titers against the SARS-CoV-2 viral proteins at day 85 were reduced 1.4 to 1.7 fold from day 29. Similar reductions were observed for the cohorts treated with mAbs: among the bamlanivimab monotherapy cohorts, least square means of titers against Spike-NTD and NCP at day 85 was reduced 1.3 to 1.5 fold and 1.6 to 1.7 fold, respectively, from day 29. Treatment with bamlanivimab and etesevimab together resulted in 1.3 and 1.6 fold reductions in least square means of titers against Spike-NTD and NCP at day 85 from day 29.

**Table 2:** Details on SARS-CoV-2 proteins.

	Serology Assays		
Protein	SARS-CoV-2 Sequence Length (AA)	Backbone	Expression
Full-length Spike (with D614G)	1195 (14-1208)	Wuhan WT spike	CHO
Spike-RBD	199 (329-527)	Wuhan RBD	CHO
Spike-RBD E484Q	223 (319-541)	Wuhan RBD	CHO
Spike-NTD	294 (14-307)	Wuhan NTD	CHO
NCP	419 (1-419)	Wuhan NCP	CHO
	**Pseudovirus Assays**		
E484Q	1256 (1-1256)	Wuhan spike	NA
E484K	1256 (1-1256)	Wuhan spike	NA
B.1.351	1253 (1-241; 245-1256)	Wuhan spike with (L18F,D80A,D215G,del242-244, K417N,E484K, N501Y,D614G, A701V)	NA

CHO, Chinese hamster ovary cells; NA, not applicable; NCP, nucleocapsid protein; NTD, N-terminal domain; RBD, receptor-binding domain; WT, wild-type.

### Effect of mAb Treatment on Endogenous Antibody Titers

**Figure 2:**
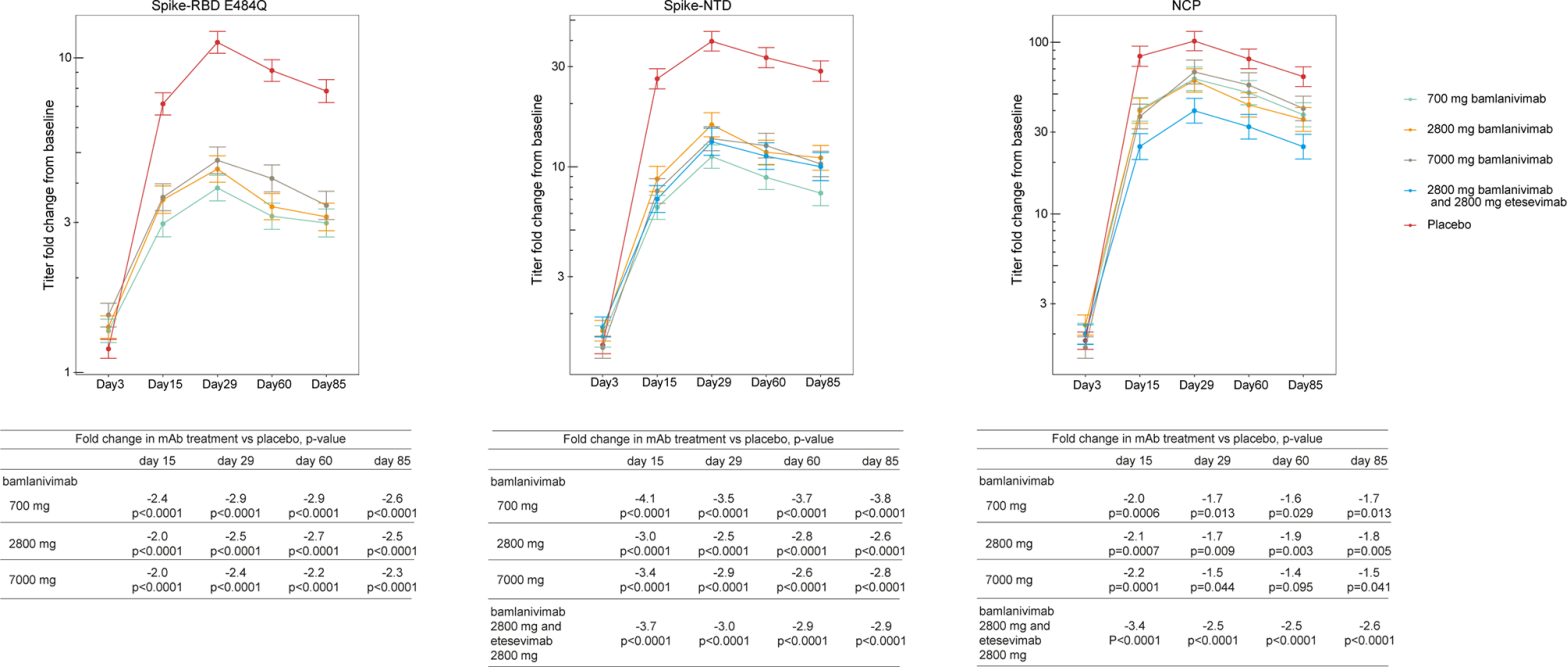
Treatment effect of bamlanivimab monotherapy or bamlanivimab and etesevimab together at days 3, 15, 29, 60 and 85. Least squares means ( ± SE) of fold changes from baseline were plotted across visit days for different treatment groups: green= 700 mg bamlanivimab, orange= 2800 mg bamlanivimab, gray = 7000 mg bamlanivimab, blue= 2800 mg bamlanivimab and 2000 mg etesevimab, and red = placebo. Titers against Spike-RBD E484Q not shown for cohort receiving bamlanivimab and etesevimab together as etesevimab binds to this mutant protein. The number of samples at each timepoint are outlined in **Table 1**. RBD, Receptor binding domain; NCP, Nucleocapsid protein; NTD, N-terminal domain; SE, Standard error.

Next, we calculated the titer change from baseline at day 3, 15, 29, 60, and 85 against the Spike-NTD and NCP (i.e., proteins that bind neither bamlanivimab nor etesevimab), as well as Spike-RBD E484Q (a mutation that negatively impacts bamlanivimab binding) (**Figure 2**). Compared to placebo, treatment with bamlanivimab monotherapy resulted in an attenuated increase in antibody titer changes from baseline from day 15 through day 85 against Spike-E484Q (ranging from 2.0 to 2.9 fold across bamlanivimab doses and time points), Spike-NTD (ranging from 2.5 to 4.1 fold), and NCP (ranging from 1.4 to 2.2 fold) (**Figure 2**). Similarly, among recipients of bamlanivimab and etesevimab administered together, an attenuated increase in antibody titer changes from baseline from day 15 through day 85 against both Spike-NTD (ranging from 2.9 to 3.7 fold) and NCP (ranging from 2.5 to 3.4 fold) were observed compared with placebo.

### mAb Treatment Effect on Neutralization of SARS-CoV-2 Pseudoviruses

**Figure 3:**
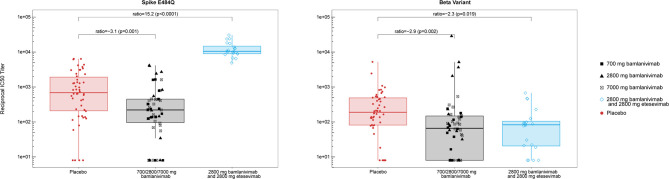
Neutralization activity of serum samples against spike E484Q and beta variant (B.1.351) at day 29. Each data point represents the reciprocal IC50 titer from serum samples collected from an individual patient. Median of the reciprocals of IC50 titers against the spike E484Q were 225, 213, and 220, for sera collected from the bamlanivimab 700, 2800, and 7000 mg cohorts, respectively. Median of the reciprocals of the IC50 titers against beta (B.1.351) were 49, 87, and 86, for sera collected from the bamlanivimab 700, 2800, and 7000 mg cohorts, respectively. Boxes and horizontal bars denote the interquartile range (IQR) and the median reciprocal IC50 titer, respectively. The whiskers are equal to the maximum and minimum values below or above the median at 1.5 times the IQR. Ratio = median in bamlanivimab or bamlanivimab and etesevimab vs median in placebo. p = p-value from non-parametric Steel’s test.

To probe the functionality of the polyclonal antibody response, we tested a randomly selected subset (stratified by treatment group) of the day 29 serum samples for neutralization activity. SARS-CoV-2 viral neutralization was measured using a vesicular stomatitis virus (VSV)-based pseudovirus (18, 28). We assessed IC50 titers of serum samples against three different pseudoviruses, containing the E484Q or E484K substitutions in spike, as well as the beta-variant (B.1.351) which contains E484K and K417N substitutions that have been shown to significantly reduce the binding of both bamlanivimab and etesevimab (29). Bamlanivimab treatment (all dose levels pooled) resulted in significantly smaller neutralization of spike E484Q pseudovirus compared with placebo (p=0.001) with the median of the bamlanivimab group 3.1-fold lower compared to the median of the placebo group. Similar neutralization activity was observed against the Spike E484K pseudovirus (data not shown). As anticipated, treatment with bamlanivimab and etesevimab together resulted in significantly increased neutralization of spike E484Q pseudovirus compared with placebo (p<0.0001) with the median of the bamlanivimab and etesevimab together group 15.2-fold higher compared to the median of the placebo group, due to the presence of etesevimab in the serum (**Figure 3**). Treatment with bamlanivimab alone or bamlanivimab and etesevimab together reduced sera neutralization against the beta variant B.1.351 compared with placebo (**Figure 3**). Reciprocal IC50 values were slightly lower in both bamlanivimab monotherapy (p=0.002) and bamlanivimab and etesevimab together (p=0.019) treatment arms compared with placebo with medians 2.9 and 2.3-fold lower than placebo median, respectively.

### Early Viral Load Predicts Antibody Titer

**Figure 4:**
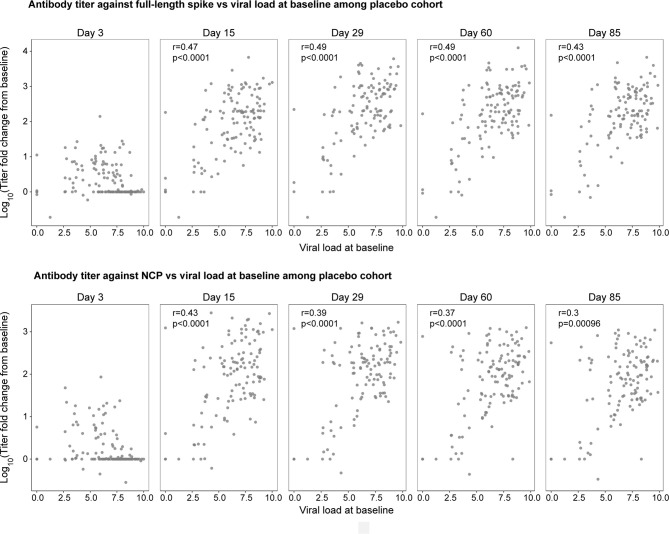
The natural history relationship between viral load at baseline (x-axis) and the fold change in antibody titer against the full-length spike (top panel) and NCP (bottom panel) from baseline to day 3, 15, 29, 60, and 85 in the placebo cohort. 42% and 54% of patients were sero-negative at both baseline and day 3 for full-length spike and NCP, respectively. r is the spearman correlation between viral load and log of titer fold change from baseline; p is the p-value associated with spearman correlation test.

Next, we explored whether the amount of SARS-CoV-2 nasopharyngeal viral load impacts the magnitude of the endogenous, pharmacologically unmodified antibody response. Viral load was measured by nasopharyngeal swab followed by quantitative reverse transcriptase–polymerase chain reaction (13). Using serum samples provided by patients in the placebo cohort, we investigated the relationship between early viral load at baseline and the log_10_ change from baseline in antibody titer against the full-length spike and NCP at days 3, 15, 29, 60, and 85 (**Figure 4**). Patients with higher viral loads at baseline showed greater fold increases in antibody titers against the full-length spike and the NCP proteins on day 15 (r=0.47, r=0.43, respectively; p<0.0001) and day 29 (r=0.49, r=0.39, respectively; p<0.0001) when antibody responses peaked (but not on day 3 when most titers were below the detection limit), indicating that baseline viral load determines the magnitude of the humoral response. These relationships between early viral load and antibody titers against the full-length spike and the NCP proteins persisted to day 60 (r=0.49, r=0.37, respectively; p<0.0001) and day 85 (r=0.43, r=0.3 respectively; p<0.001). Similar relationships were observed between antibody titers and viral loads at day 3 (data not shown).

## Discussion

This study provides a comprehensive assessment of the effects of mAb treatment on endogenous antibody responses to SARS-CoV-2 infection.

We found that patients produce a wide breath of serological responses against SARS-CoV-2 epitopes regardless of mAb treatment, with responses durable through to 85 days. To explore the effects on endogenous responses among mAb recipients, we utilized the finding that SARS-CoV-2 variants carrying mutations in the RBD of the spike protein have the potential to evade mAb treatment (15, 23, 29, 30). As the E484 residue is a key contact within the epitope of bamlanivimab (25), E484K and E484Q substitutions greatly attenuate binding of bamlanivimab; however, binding of etesevimab persists, due to its distinct binding epitope (13). Therefore, we examined changes in antibody titers against the Spike-RBD E484Q protein, in addition to titers against proteins that lie outside of the epitopes of both bamlanivimab and etesevimab (Spike-NTD and NCP). We found that mAb treatment resulted in smaller increases in anti-SARS-CoV-2 endogenous antibody titers (1.4 to 4.1 fold) as compared to placebo, with some being statistically significant. Despite these small reductions, antibody titers among mAb recipients showed comparable patterns to the placebo group from baseline to day 85. Clinical relevance of these slightly lower titers is not known. Further, it remains to be seen whether mAb-treated patients and placebo-treated patients possess equal levels of protection upon SARS-CoV-2 re-exposure. The effect of mAb treatment on memory responses (including T and B cells) also remains unknown. Importantly, the level of exogenously administered antibody is much higher than the titer of endogenous anti-spike antibodies, so patients who receive mAb treatment have an overall greater ability to neutralize virus.

To assess the effects of mAb treatment on the spectrum of epitopes neutralized, we measured IC50 titers against Spike E484Q, Spike E484K, and the beta variant (B.1.351). The latter is a variant of concern and has two key mutations in the RBD of spike, E484K and the K417N, and can escape both bamlanivimab and etesevimab recognition and neutralization *in vitro* (15, 31). We found that serum samples collected from patients who received bamlanivimab treatment were slightly less effective in neutralizing spike E484Q and beta variant compared with placebo (by a factor of 3.1 and 2.9, respectively). As noted before, it is unknown whether these small reductions are clinically meaningful.

Finally, we investigated the relationship between the early viral load and endogenous antibody titers over time within the placebo cohort. The results show that individuals with lower viral loads at baseline generate lower antibody titers at later time points (i.e. from day 15 and beyond), suggesting that early viral load determines the magnitude of the subsequent antibody response. This finding, taken together with findings from previous reports showing that mAb treatment administered early during infection reduces SARS-CoV-2 viral load (13, 19), suggest that the reductions in endogenous antibody titers among mAb recipients may be due to reduced early viral load as a result of the efficacy of mAb treatment. In addition, bamlanivimab is an IgG1 and may also inhibit endogenous B cell activation through engagement of FcγRIIb, though we do not know the level of contribution of this relative to the effect of decreased viral antigen exposure. As mean viral load at baseline was similar across treatment groups (13), the observed effects on endogenous antibody responses can be attributed to mAb-dependent reductions in viral load following treatment. Importantly, this reduction in endogenous antibody responses does not slow viral clearance during acute SARS-CoV-2 infection; indeed, viral decay is significantly accelerated among patients administered bamlanivimab alone or bamlanivimab and etesevimab together (13, 19). This finding may have implications for optimal timing of SARS-CoV-2 vaccination following recovery from COVID-19 as a result of neutralizing mAb therapy.

Taken together, the similarity in breadth and duration of response between the placebo and mAb treatment cohorts suggest that a similar immune response was induced upon SARS-CoV-2 infection, but that the magnitude of endogenous antibody production was attenuated presumably due to the reduction in antigen exposure (as suggested by reduced viral load) achieved by mAb treatment.

This study has several limitations. First, it is not yet known whether the risk to reinfection with SARS-CoV-2 is similar for mAb-treated patients and placebo-treated patients. Second, since mAb treatment resulted in a small effect on endogenous antibody production, we hypothesize that treatment may also impact vaccine-induced antibody responses. However, this remains to be evaluated in future dedicated studies. Third, we could not evaluate changes to antibody titers for additional SARS-CoV-2 variants, nor wild-type spike/RBD proteins in this study due to drug interference. Other groups have found that the polyclonal immune response for some individuals has at least a proportion that is directed at the E484 position (32). By using the drug tolerant E484Q/K spike-RBDs in this investigation, we may be underestimating the overall endogenous spike immune response. Fourth, we assessed the impact of just two mAbs (bamlanivimab alone or bamlanivimab and etesevimab together) on the endogenous immune response, and therefore we do not know the effect of other mAbs on the immune response. However, we hypothesize similar results, as those mAbs also reduce viral load upon administration (33).

In conclusion, this research identified that mAb therapy for COVID-19 infection does not abolish the endogenous immune response against SARS-CoV-2, but instead results in only minor attenuations of titer and neutralization capacity. We hypothesize that these minor changes pose very low risk for patients in terms of reinfection and long-term immune protection.

## Data Availability Statement

The original contributions presented in the study are included in the article/supplementary material. Further inquiries can be directed to the corresponding author.

## Ethics Statement

This study (BLAZE-1 ClinicalTrials.gov number, NCT04427501) was conducted in accordance with the Declaration of Helsinki and Council for International Organizations of Medical Sciences International Ethical Guidelines, and applicable International Council for Harmonization Good Clinical Practice Guidelines, laws, and regulations. The protocol was reviewed and approved by the ethics committees of all participating centers, and patients provided written informed consent before study entry.

## Author Contributions

LZ, JP, RLG, and RB conceptualized the study. LZ, JP, MDo, SB, TN, MC, JF, PV, DP, AN, NK, and RB contributed to the study design. JP, MDo, PC, GH, RLG, SB, MDa, TNVT, MC, and DP contributed to data acquisition. LZ, JP, GH, MD, RLG, SB, MDa, DP, AN, NK, RH, and RB contributed to data interpretation. LZ, JP, RLG, MDa, SB, TN, MC, and RH analyzed the data. LZ, JP, MDo, PV, NK, and RB contributed to the writing of this paper. All authors revised the manuscript. All authors read and approved the final version for submission.

## Funding

This work was supported by Eli Lilly and Company.

## Conflict of Interest

Authors LZ, JP, SB, MDa, TNVT, MC, JF, PV, DP, AN, NK, RH, and RB are all employees and shareholders of Eli Lilly and Company. MDo reports receiving grants from Eli Lilly and Company, and Novartis; and receiving consulting fees from Moderna, Tillotts, SQZ, AzurRx, Partner Therapeutics, and ORIC Pharmaceuticals; and has Neoleukin Therapeutics stock. PC reports receiving consulting fees from Eli Lilly and Company and Gilead; and receiving payment or honoraria from Rockpointe, Frontier Collaborative, CME Outfitter, and Physician Education Resource. RG reports receiving consulting fees from Eli Lilly and Company, GSK Pharmaceuticals, and Gilead Sciences; and receiving payment or honoraria from Gilead Sciences. GH reports receiving grants from Eli Lilly and company, Gilead, Viiv, Janssen, and Proteus; receiving consulting fees from Gilead, Viiv, and Janssen; and receiving payment or honoraria from Rockpointe, CME Outfitter, Simply Speaking, and Clinical Care Options.

The authors declare that this study received funding from Eli Lilly and Company. The funder had the following involvement with the study: study design, data analysis, decision to publish, and preparation of the manuscript.

## Publisher’s Note

All claims expressed in this article are solely those of the authors and do not necessarily represent those of their affiliated organizations, or those of the publisher, the editors and the reviewers. Any product that may be evaluated in this article, or claim that may be made by its manufacturer, is not guaranteed or endorsed by the publisher.
